# Assessment of Various Risk Factors for Biological and Mechanical/Technical Complications in Fixed Implant Prosthetic Therapy: A Retrospective Study

**DOI:** 10.3390/diagnostics13142341

**Published:** 2023-07-11

**Authors:** Dimitrios Bardis, Doriana Agop-Forna, Stavros Pelekanos, Nicolae Chele, Cristina Dascălu, Roland Török, Bianca Török, Ioana Cristea, Panagiota Moulavasili Bardi, Norina Forna

**Affiliations:** 1 Faculty of Dental Medicine, Grigore T. Popa University of Medicine and Pharmacy Iasi, Universitatii Street 16, 700115 Iasi, Romania; dimbardis@gmail.com (D.B.); cristina.dascalu@umfiasi.ro (C.D.); cristeaioanadent@gmail.com (I.C.); norina.forna@umfiasi.ro (N.F.); 2 Independent Researcher, Leof. El. Venizelou 163b, 176 72 Athens, Greece; 3 Faculty of Dental Medicine, U.S.M.F. “Nicolae Testimitanu”, Stefan cel Mare si Sfant Boulevard 165, 2004 Chisinau, Moldova; nicolae.chele@usmf.md; 4 Independent Researcher, 1712 Tafers, Switzerland; roland.toeroek@gmx.de (R.T.); drbianca.toeroek@gmx.de (B.T.); 5 Independent Researcher, Ippokratous 166, 114 71 Athens, Greece; panbardi@gmail.com

**Keywords:** dental implants, risk factors, implant failure, implant-supported fixed partial dentures, periodontal disease, peri-implantitis, prosthetic success

## Abstract

The goals of this research were to determine the influence of several factors on implants’ biological and technical complications in posterior fixed implant prosthetic therapy. Materials and methods: The study group consisted of 67 edentulous patients (mean age: 63.88 ± 11.709 yrs; 20 males, 47 females) with implant prosthetic therapy for posterior edentulism. A total of 76 implant-supported fixed partial dentures (IP-FPDs) and 178 implants were assessed using clinical and paraclinical assessments. Risk factors for biological complications (peri-implantitis) and technical complications were determined by using the Pearson Chi-squared test and multivariate analysis. Results: The implant success (the absence of biological and mechanical/technical complications) was 66.30%. The prevalence of biological complications was 13.5%. The prevalence of technical complications was 28.70%. Variables that were associated with a higher risk of peri-implantitis were poor oral hygiene and bruxism. In univariate analysis, poor oral hygiene increased the risk of peri-implantitis 5.778 times and bruxism 5.875 times. Variables that were associated with a higher risk of mechanical/technical complications were age group > 60 yrs, smoking, history of periodontal disease, and bruxism. In univariate analysis, the risk of technical complications increased 4.14 times for patients in the age group > 60 years (vs. age group 40–60 years) and 20.5 times for patients with bruxism. Bruxism and smoking were significant predictors of mechanical/technical complications in the multivariate model. Conclusions: In univariate models, patients with poor oral hygiene and bruxism have an increased risk of peri-implantitis. In multivariate models, we did not identify significant predictors of peri-implantitis. Age group > 60 yrs, smoking, history of periodontal disease, bone grafting, and bruxism are risk factors for the increase in the mechanical/technical complication rate. In the multivariate model, smoking and bruxism are significant predictors of the mechanical/technical complications.

## 1. Introduction

The long-term dental implant prognosis is determined by, among other factors, the assessment of the risk factors in the planning stage and by stabilizing or removing preexisting oral diseases prior to the start of implant surgery. In this context, the outcome of implant prosthetic treatment is influenced by various factors as follows: implant-related factors (previous implant failure, implant surface status, degree of exposure to the oral environment); mechanical factors (premature loading, occlusal trauma); patient-related factors (level of oral hygiene, periodontal tissue condition, peri-implant bone condition, distance to adjacent teeth, periodontal status of adjacent teeth, soft tissue condition); systemic factors (smoking, age-related pathology, nutritional deficiencies, diabetes, steroid therapy, chemotherapy or radiotherapy); and surgical technique factors [1,2,3,4,5,6,7,8,9].

To reduce the risk of the implant failures due to biological or mechanical/technical complications, risk factors must be eliminated or ameliorated. Patients’ compliance with a strict follow-up protocol, including consistent home care and maintenance sessions in a private dental office, plays an important role in the early diagnosis of the inflammatory peri-implant processes as well as early detection of factors influencing the onset of the mechanical or technical complications [2,3].

The goal of this research was to determine the influence of several factors on the biological and technical complications in fixed implant prosthetic therapy of posterior edentulism.

## 2. Materials and Method

The research was performed according to the ethical values of the Declaration of Helsinki and received approval from the ethics committee of U.M.F. “Grigore T.Popa” Iasi (Romania) (No.19355). All patients were informed about the research objectives and provided written informed consent.

### 2.1. Study Design and Inclusion and Exclusion Criteria

This was a retrospective study including 67 patients (mean age: 63.88 ± 11.709 yrs; gender: 20 males, 47 females) with posterior partial edentulism that were treated in a private practice from 2006 to 2018. The implant prosthetic therapy was performed with Nobel Biocare HQ (Kloten, Switzerland) implants (178 implants; length 10–13 mm; width 3.5–4.5 mm) and metal–ceramic implant-supported fixed partial dentures (IP-FPDs). The mean follow-up time was 7.89 ± 4.626 yrs. The subjects were selected from patients invited to recall. The inclusion criteria were as follows: age ≥ 18 years; 3–5 units IP-FPDs with centric pontic; follow-up 3–15 years. Exclusion criteria were decompensated metabolic diseases and non-compliant patients to periodontal maintenance visits.

The design of the study followed the PICO components (Table 1).

The features of the study group at recall are presented in Table 2, globally, as well as by comparison according to the implants’ survival.

### 2.2. Implant Stage and Prosthetic Procedures

Before the implant stage, patients with periodontal pathology were treated by non-surgical or surgical procedures by a periodontologist. A single surgeon (P.M.B.), with >15 years of experience, performed the alveolar bone grafting procedures and the implant surgical technique. The alveolar bone rehabilitation was required for 49.4% of the implant sites. For patients with severe resorbed alveolar bone, the implant site’s reconstruction was performed by horizontal and vertical augmentation techniques with xenografts and resorbable collagen membranes [10,11]. Implants were placed following standard implant protocol by delayed implant placement protocol [12,13]. A chlorhexidine 0.12% rinse was recommended pre- and post-operatively. Patients that underwent alveolar bone addition procedures received a preoperative loading dose of antibiotics and postsurgical doses for 3–5 days in relation to infectious risk [14]. Implants were conventionally loaded (delayed loading) at 3 months with definitive metal–ceramic fixed dentures [15,16].

### 2.3. Definitions

The definition of implant survival is as follows: implant and abutment still present in the mouth at follow-up examination [4].

The definition of implant failure is as follows: implants that require removal or have already been lost [4]. ICOI introduced failed implants in the Group IV Pisa Implant Health Scale that includes both implants unable to be restored and implants associated with any of these conditions: (1) palpation, percussion, or function associated with pain, (2) horizontal and/or vertical mobility, (3) severe and progressive bone loss, (4) uncontrolled exudate, or (5) more than 50% bone loss around the implant [4].

The definition of success is as follows: presence of the implant, abutment, and prosthetic suprastructure in situ without biological complications [6] or without mechanical or technical complications during the follow-up time [5].

Implants with biological complications were considered implants with peri-implantitis (associated with progressive marginal loss) [9,17], while peri-mucositis is a reversible condition under proper treatment [17]. Criteria for a peri-implantitis diagnosis were as follows [18,19,20,21]:

- Peri-inflammation signs at the clinical level (erythema, swelling, bleeding on probing, and/or suppuration);
- In the absence of a previous radiograph, radiologic bone loss of at least 3 mm (from the implant shoulder) combined with probing depth of ≥6 mm associated with BOP.

The mechanical complications are the loss of screw hole access material, screw loosening, abutment loosening, screw fracture, or implant fracture [9,18]. The technical complications of the implant-supported fixed partial dentures are fracture/chipping of veneering ceramic and fracture of the framework of fixed partial dentures [9,18].

### 2.4. Clinical Examination

Complete clinical examinations were performed by one independent and calibrated examiner (DB) from October 2021 to May 2022. The following categories were assessed:

- Medical history;
- Familial history;
- Smoking history (smokers > 10 cigarettes/day; non-smokers);
- Mechanical/technical complications: absent, major complications such as implant fracture, medium and minor complications (fracture of abutment, veneer or framework, veneering chipping, loosening of abutment or screw, loss of retention);
- Peri-implant soft tissue condition: probing pocket depth (PPD) using a manual periodontal probe (Click-Probe^®^, Kerr, Bioggio, Switzerland) at six sites per implant, bleeding on probing (BOP), mobility;
- Modified gingival index (mGI) [22];
- Modified plaque index (mPII) at all implants [22];
- Width of keratinized tissue (mm) (mandible: 6 sites/implant; maxillary: 3 sites/implant)—measured from the mucogingival junction and the most coronal point of the keratinized mucosa in the center of the IP-FPD.

### 2.5. Radiologic Analysis

The CBCT exam (Sirona Orthophos XG - Charlotte, North Carolina, United States of America) was used to calculate the peri-implant marginal bone loss (MBL). CBCT scanning conditions were the following: 85 kV, 6 mA, 14.4 s irradiation time, 25–1025 µSv irradiation dose, 1 mm slice thickness. The measurements were made by an independent radiologist who was not involved in the study. At the mesial and distal implant sides, Sidexis XG/DVT (Densply/Sirona) software was used to measure the distance between the connection implant abutment and the level of MBL. The highest value was taken as the extent of bone loss.

### 2.6. Data Collection

All patients were examined during the yearly regular visit for implant and surrounding hard and soft tissue status. Data were collected and introduced into an SPSS database by one investigator (DB). The following data were collected: gender and age group (40–60 yrs vs. >60 yrs), oral hygiene (mPI index), as well as data regarding biological complications and mechanical/technical complications.

### 2.7. Statistical Analyses

The statistical analyses were performed in SPSS 29.0. The qualitative variables were characterized through frequency distributions. The quantitative variables were characterized through descriptive statistics (averages and standard deviations). The assessed variables were as follows: demographic factors (age group: 40–60 yrs vs. >60 yrs; gender), smoking (1–10 cigarettes/day vs. no smoking), history of periodontal disease (present vs. absent), oral hygiene (mPI 0–1 vs. 2–3), bruxism (present vs. absent), implant number (2 vs. 3), bone grafting (present vs. absent), follow-up (3–5 yrs vs. 6–10 yrs vs. >10 yrs), edentulism location (Mx vs. Md vs. Mx + Md), opposing arch (natural teeth vs. removable dentures with implant support vs. metal–ceramic fixed dentures with implant support vs. natural metal–ceramic fixed dentures with implant support). The risk factors for the biological and mechanical/technical complications were assessed through the Chi-squared test and OR (Odds Ratio), as well as through binary logistic regression. The survival time was evaluated through Kaplan–Meyer analysis and Log-Rank (Mantel–Cox) test. The degree of statistical significance was set at *p* < 0.05.

## 3. Results

### Implant Data at Recall and Implant Survival and Success Rates

The implant data at recall (PPD; mean MBL; BOP; mGI; mPI; width of keratinized mucosa) are shown in Table 3.

The implant survival rate was 96.6% (172 implants from the total of 178). The implant success rate (absence of biological/technical complications) was 66.3% (118 implants from the total of 178). The complications reported at the level of the 178 implants were as follows: peri-implantitis (24 cases—13.5%); mechanical/technical complications (51 cases—28.7%).

Table 4 exposes the univariate analysis on peri-implantitis in relation to variables such as smoking, history of periodontal disease, poor oral hygiene, bruxism, follow up > 10 years, and FPD (metal–ceramic) with natural teeth support on the opposing surfaces (Table 4).

The binary logistic regression model of the significant risk factors is statistically significant (*p* < 0.001—Omnibus Test of Model Coefficients) and explains 69.1% of the variance of peri-implantitis (Nagelkerke R2), with a sensibility of 87.5% and a specificity of 100.0%; the identified statistically significant predictors were the history of periodontal disease and implant site grafting (Table 5).

The risk factors for mechanical/technical complications, as depicted in Table 6, are age group > 60 yrs, smoking, history of periodontal disease, poor oral hygiene, bruxism, support of two implants, implant site grafting, follow-up at 3–5 years but also at over 10 yrs, mandibular location, and opposing surfaces on FPD (metal–ceramic) with teeth support (Table 6).

The binary logistic regression model of the significant risk factors is statistically significant (*p* < 0.001—Omnibus Test of Model Coefficients) and explains 69.1% of the variance of technical complications (Nagelkerke R2), with a sensibility of 82.4% and a specificity of 97.6%; the identified statistically significant predictors were the history of periodontal disease, bruxism, FPD with support of two implants, mandibular edentulism, and opposing surfaces on FPD (metal–ceramic) with natural teeth support (Table 7).

The average survival time of the dental implants is presented in Table 8 (follow-up 3–17 years). The implants’ average survival time was 16.528 years, while the survival time without biological or technical complications was 12.471 years.

## 4. Discussion

Data regarding the risk factors for implant success are scarce, despite the increasing body of evidence-based knowledge regarding the prevalence of biological and mechanical/technical complications. Experienced dental practitioners must plan the implant prosthetic treatment based on proper long-term expectations of implant success and must consider possible future biological and mechanical/technical complications when preparing patients for receiving their informed consent [23].

In our study, the definitions of the implant survival and success were based on the statement of the International Congress of Oral Implantologists (ICOI) Consensus Conference for Implant Success (2007) [4]. In fixed implant prosthetic therapy, the major role of the dental implants is to act as abutments for fixed restorations, similar to a natural tooth. In this context, ICOI stated that any success criteria must include the implants’ ability to support functional dentures [4]. However, implant success is difficult to describe in the same way as the success criteria required for a tooth [4]. We considered implant success as only those implants that were not associated with major biological complications (peri-implantitis) or any technical and mechanical complications during the follow-up time [5]. This category of implants corresponds to Group I of the Pisa Implant Health Scale with very good to excellent prognosis (the absence of any biological or technical complications as well as the lack of association with mechanical complications of their prosthetic suprastructure). Despite the stability and the absence of symptoms (pain, tenderness) (Group II of the Pisa Implant Health Scale), the presence of the peri-implantitis has a potential for early clinical problems [4]. Moreover, implants exhibiting a slight-to-moderate peri-implantitis and compromised health status (Group III of the Pisa Implant Health Scale) are implants that can be associated with technical or mechanical complications of its prosthetic suprastructure.

The goal of our study was to highlight several risk factors for complications (biological, mechanical/technical) associated with implant poor prognosis or failure. Our research included only patients who were compliant to the annual maintenance sessions. The implant-prosthetic therapy outcomes require an assessment made through patient-based parameters, as the patient becomes central in the overall analysis. In this context, the treatment success is not based only on the clinical and technical aspects but also on the compliance of patients with oral hygiene rules and maintenance sessions [24]. The literature has a great variability of data regarding both the prevalence of implant success and biological and mechanical/technical complications due to different criteria systems and evaluation protocols and techniques. Studies with a minimum of 5-year follow-up report variable results on the prevalence of peri-implantitis. The prevalence of peri-implantitis at the implant level was 7.3% [25], 9.1% [26], 9.8% [27], 9.83% [28], 9.6% [29], 16% [30], 23% [31], 24.9% [32], and 72% [33]. At the patient level, the prevalence of peri-implantitis was 13.3% [34], 15.1% [25], 16.3% [26], 18% (patients compliance to maintenance periodontal sessions) [34], 18.8% [29], 19.83% [28], 26% [30], 37% [31], and 45% [32]. Derks & Tomasi (2015) reported the incidence of peri-implantitis of 1–47% (weighted mean prevalence of 22%) [35].

Epidemiological studies addressing biological complications are mainly aiming at the assessment of incidence and prevalence but are less focused on determining the peri-implantitis stages due to the absence of consistent case definitions, cohorts with a significant number of subjects, and longer monitoring periods to allow the interpretations of a significant percentage of biological complications [36]. There are a relevant number of long-term studies aiming to determine the prevalence of periodontitis and peri-implantitis as well as potential risk indicators or significant predictors (patient age, age of implants, periodontal status, level of oral hygiene) [23,24,25,30,31,32,35,37,38,39,40].

We found that patients’ lack of compliance with proper oral hygiene, as well as bruxism, were factors significantly associated with the onset of peri-implantitis. Poor oral hygiene was strongly correlated with the onset of peri-implantitis, while the history of periodontitis was highlighted as a risk indicator for peri-implantitis [40]. The history of periodontitis and the quality of oral hygiene were also proposed as risk factors by other studies [32,41,42]. Significant predictors of peri-implantitis were found the maxillary implant location (2.98 times higher probability of peri-implantitis when compared to the mandibular area) and age group < 60 years [33].

The frequency of peri-implantitis increases significantly if the mean follow-up is more than 8 years [30]. A positive correlation was detected between peri-implantitis and the parameters age, history of periodontal disease, and the number of missing teeth [31]. Increased plaque index increases the probability of peri-implantitis by 1.36 times, while the use of the alveolar augmentation techniques of the implant site reduces the risk of peri-implantitis (OR = 0.87); the loss of a tooth due to periodontal disease increases the risk of peri-implantitis (OR = 1.063) as well as the maxillary location of the implants are associated with increased probability of peri-implantitis (OR = 1.052) [31]. The authors of the study concluded that the low level of oral hygiene and active periodontal disease represent the most significant risk factors for the occurrence of peri-implantitis [31]. In our study, age group, gender, and implant location were not found as significant predictors of peri-implantitis; this result is confirmed by a research that failed to correlate these factors with implant failure [43]. We found that smokers has 6.11 times higher risk of peri-implantitis when compared to non-smokers. One study reported that smoking was significantly correlated with peri-implantitis prevalence [43]. Increased prevalence of peri-implantitis was higher in smokers (36.3%) while the maintenance periodontal therapy has a significant role in reducing the risk of peri-implantitis [29]. The prevalence of moderate/severe peri-implantitis was higher in patients with fixed implant-prosthetic restorations with follow-up > 9 years [32]. However, the peri-implant pathology is not the only factor that induce marginal bone loss. Other reasons include physiological remodeling after implant insertion, occlusal overload, practitioner experience in surgical and prosthetic stages, level of oral hygiene, and systemic status [44,45]. 

Peri-implantitis is considered a major complication with relevant impact on the implants’ survival and success. However, it must not be underestimated the role of the mechanical and technical complications in the long-term outcome of implant therapy. In our current study, we found a higher risk of mechanical/technical complications for patients in the age group > 60 years, smokers, and patients with a history of periodontal disease or bruxism. In multivariate analysis, the most significant predictors for mechanical/technical complications were smoking and bruxism. Bredberg et al (2023) found a significant association between the combination of bruxism and smoking and peri-implant increased marginal bone loss for patients with minimum 36 months follow-up. Patients who are both bruxers and smokers had significantly greater marginal bone loss when compared to patients who are either a bruxer or smoker, or neither [46]. We found that patients with bruxism have a 15.23 times higher risk of mechanical/technical complications when compared to non-bruxers. Chrchanovic also reported a significant higher risk of implant failure associated to mechanical/technical complications (OR 2.71) [47]. Despite significantly correlation of bruxism with mechanical/technical complications, other risk factors must be considered and analyzed in further studies. Our results regarding the prevalence of mechanical/technical complications are in range of those reported by literature data [48,49,50]. We found that age group > 60 yrs., smoking, history of periodontal disease, bone grafting, and bruxism are associated with the increase of the mechanical/technical complications rate. The most frequent mechanical/technical complications reported for implant-supported FPDs located in posterior areas are as follows: loss of screw access whole material (23.6%), followed by ceramic veneers fracture/chipping (11.8%), and screw loosening (8.4%) [51]. Bäumer et al (2020) found a 19.4% rate of implant technical complications, with abutment/screw loosening being the most common complication (5.3%) [52]. Literature data associated higher rates of the mechanical/technical complications with excessive implant loading, bruxism, the length of the implant-prosthetic reconstruction, and a history of repeated complications [5,53]. Some authors propose laser or photodisinfection as a means of treatment for peri-implantitis and this could constitute a solution for such compromised cases [54,55].

Some limitations of the current study must be considered: retrospective design, patients selected from standard pool of private dental practice, and a homogeneous study group consisting of patients recruited from a single private dental practice. However, the implant survival and success rates reported by our study were in range of those reported by literature data. Different criteria and definitions of implant survival, success, and failure may explain the differences in the implant prosthetic success between various research groups, which contribute to the limitation of reliable interpretations and direct comparisons of data between studies [4]. The research groups highlighted some of the specific protective factors that allow the decrease of the rate of mechanical/technical complications (changes in implant design, optimized implant dimensional parameters, progressive loading, immediate implantation techniques, optimized design of the implant-supported prosthetic restorations) [4]. 

## 5. Conclusions

In univariate models, patients with poor oral hygiene and bruxism have an increased risk of peri-implantitis. In multivariate models, significant predictors of peri-implantitis were not identified. Age group > 60 years, smoking, history of periodontal disease, bone grafting, and bruxism are risk factors for the increase in the implant mechanical/technical complication rate. In the multivariate model, bruxism is a significant predictor of mechanical/technical complications.

## Figures and Tables

**Table 1:** Study design (PICO) components.

Component	Description
Population (P)	Patients with posterior edentulism
Intervention (I)	Fixed implant prosthetic therapy—3–5 units metal–ceramic IP-FPDs
Comparison (C)	Implants with biological complications (peri-implantitis) Implants associated with mechanical/technical complications
Outcome (O)	Risk factors for biological complications (OR) Risk factors for technical complications (OR)

**Table 2:** Study group features (at recall).

		Implants’ Survival		
	Total (*n* = 178)	Yes (*n* = 172) (100.0%)	No (*n* = 6) (100.0%)	*p*
Age group				0.190
40–60 yrs	49 (27.5%)	49 (28.5%)	0 (0.0%)	
>60 yrs	129 (72.5%)	123 (71.5%)	6 (100.0%)	
Gender				0.179
M	55 (30.9%)	55 (32.0%)	0 (0.0%)	
F	123 (69.1%)	117 (68.0%)	6 (100.0%)	
Smoking status				0.193
Non-smoker	130 (73.0%)	124 (72.1%)	6 (100.0%)	
Smoker	48 (27.0%)	48 (27.9%)	0 (0.0%)	
History of periodontal disease				0.340
Yes	45 (25.3%)	45 (26.2%)	0 (0.0%)	
No	133 (74.7%)	127 (73.8%)	6 (100.0%)	
Oral hygiene (mPI)				1.000
0–1	157 (88.2%)	151 (87.8%)	6 (100.0%)	
2–3	21 (11.8%)	21 (12.2%)	0 (0.0%)	
Bruxism				0.592
Yes	31 (17.4%)	31 (18.0%)	0 (0.0%)	
No	147 (82.6%)	141 (82.0%)	6 (100.0%)	
Implant number/FPD				0.036 *
2	100 (56.2%)	94 (54.7%)	6 (100.0%)	
3	78 (43.8%)	78 (45.3%)	0 (0.0%)	
Implant site grafting				0.029 *
Yes	88 (49.4%)	88 (51.2%)	0 (0.0%)	
No	90 (50.6%)	84 (48.8%)	6 (100.0%)	
Follow-up (years)				0.026 *
3–5 yrs	82 (46.1%)	76 (44.2%)	6 (100.0%)	
6–10 yrs	48 (27.0%)	48 (27.9%)	0 (0.0%)	
>10 yrs	48 (27.0%)	48 (27.9%)	0 (0.0%)	
Edentulism location				0.029 *
Mx	91 (51.1%)	85 (49.4%)	6 (100.0%)	
Md	87 (48.9%)	87 (50.6%)	0 (0.0%)	
Implant location				0.553
C/IL	15 (8.4%)	15 (8.7%)	0 (0.0%)	
PM	58 (32.6%)	55 (32.0%)	3 (50.0%)	
M	105 (59.0%)	102 (59.3%)	3 (50.0%)	
Opposing surfaces				0.015 *
Natural teeth	58 (32.6%)	58 (33.7%)	0 (0.0%)	
Removable dentures (acrylic teeth) with implant support	18 (10.1%)	18 (10.5%)	0 (0.0%)	
FPD (metal–ceramic) with natural teeth support	66 (37.1%)	60 (34.9%)	6 (100.0%)	
FPD (metal–ceramic) with implant support	36 (20.2%)	36 (20.9%)	0 (0.0%)	

* Statistically significant.

**Table 3:** Implant data at recall.

*n* = 178 (100%)	
PPD	≤3 mm: 160 (89.33%); 3.5–5 mm: 12 (6.74%); >5 mm: 7 (3.93%)
MBL (mean)	1.3625 mm (mesial); 1.0875 mm (distal)
BOP_+_ (at least 1 positive site/implant)	117 (64.74%)
mGI (max./implant)	0:68 (38.20%); 1: 85 (47.75%); 2: 18 (10.11%); 3: 7 (3.94%)
mPI (max./implant) Width of keratinized mucosa	0: 88 (49.45%); 1: 75 (42.13%); 2: 8 (4.49%); 3: 7 (3.93%) Absent: 18 (10.10%); <2 mm: 84 (47.20%); ≥2 mm: 76 (42.70%)

PPD = periodontal pocket depth; GI = modified gingival index; PI = modified plaque index; BOP = bleeding on probing.

**Table 4:** Peri-implantitis occurrence—univariate analysis results.

		Peri-Implantitis		*p*
		Yes (*n* = 24)	No (*n* = 154)	
Age group	40–60 yrs	6 (25.0%)	43 (27.9%)	0.766
	>60 yrs	18 (75.0%)	111 (72.1%)	
Gender	M	6 (25.0%)	49 (31.8%)	0.501
	F	18 (75.0%)	105(68.2%)	
Smoking status	Yes	15 (62.5%)	33 (21.4%)	0.000 **
	No	9 (37.5%)	121 (78.6%)	
History of periodontal disease	Yes	21 (87.5%)	24 (15.6%)	0.000 **
	No	3 (12.5%)	130 (84.4%)	
Oral hygiene (mPI)	0–1	15 (62.5%)	142 (92.2%)	0.000 **
	2–3	9 (37.5%)	12 (7.8%)	
Bruxism	Yes	6 (25.0%)	25 (16.2%)	0.383
	No	18 (75.0%)	129 (83.8%)	
Implant number/FPD	2	15 (62.5%)	85 (55.2%)	0.502
	3	9 (37.5%)	69 (44.8%)	
Implant site grafting	Yes	21 (87.5%)	67 (43.5%)	0.000 **
	No	3 (12.5%)	87 (56.5%)	
Follow-up (yrs)	3–5	3 (12.5%)	79 (51.3%)	0.001 *
	6–10	12 (50.0%)	36 (23.4%)	
	>10	9 (37.5%)	39 (25.3%)	
Edentulism location	MX	18 (75.0%)	73 (47.4%)	0.012 *
	MD	6 (25.0%)	81 (52.6%)	
Implant location	C/IL	3 (12.5%)	12 (7.8%)	0.071
	PM	12 (50.0%)	46 (29.9%)	
	M	9 (37.5%)	96 (62.3%)	
Opposing surfaces	Natural teeth	0 (0.0%)	58 (37.7%)	0.000 **
	Removable dentures (acrylic) with implant support	0 (0.0%)	18 (11.7%)	
	FPD with natural teeth support (ceramic)	24 (100.0%)	42 (27.3%)	
	IP-FPD (ceramic)	0 (0.0%)	36 (23.4%)	

* Statistically significant, ** Highly Statistically significant.

**Table 5:** Risk factors for peri-implantitis.

	Univariate Analysis				Multivariate Analysis			
Parameters	Pearson Chi-Squared	*p*	Risk of Peri-Implantitis		Binary Logistic Regression			
			OR/RR	95% CI OR/RR *	B Coef.	OR	95% CI OR	*p*
Smoker	17.785	0.000 **	6.111	2.456 ÷ 15.207	0.846	-	-	0.376
History of periodontal disease	56.850	0.000 **	37.917	10.482 ÷ 137.152	5.065	158.442	22.663 ÷ 1107.699	0.000 **
Oral hygiene (mPI 2-3)	17.611	0.000 **	7.100	2.573 ÷ 19.590	0.496	-	-	0.745
Implant site grafting	16.077	0.000 **	9.090	2.602 ÷ 31.756	3.280	26.585	2.863 ÷ 246.853	0.004 **
Follow-up (yrs)	13.384	0.001 *	-	-	−0.506	-	-	0.561
Maxillary location	6.329	0.012 *	3.329	1.254 ÷ 8.839	1.063	-	-	0.217
Opposing arch	47.074	0.000 **	-	-	0.217	-	-	0.627
Constant					−7.229	0.001		0.000

* Statistically significant, ** Highly Statistically significant.

**Table 6:** Mechanical/technical complications occurrence—univariate analysis results.

		Mechanical/Technical Complications		*p*
		Yes (*n* = 51)	No (*n* = 127)	
Age group	40–60 yrs	6 (11.8%)	43 (33.9%)	0.003 **
	>60 yrs	45 (88.2%)	84 (66.1%)	
Gender	M	21 (41.2%)	34 (26.8%)	0.060
	F	30 (58.8%)	93 (73.2%)	
Smoking status	Yes	6 (11.8%)	42 (33.1%)	0.004 **
	No	45 (88.2%)	85 (66.9%)	
History of periodontal disease	Yes	33 (64.7%)	12 (9.4%)	0.000 **
	No	18 (35.3%)	115 (90.6%)	
Oral hygiene (mPI)	0–1	39 (76.5%)	118 (92.9%)	0.002 **
	2–3	12 (23.5%)	9 (7.1%)	
Bruxism	Yes	24 (47.1%)	7 (5.5%)	0.000 **
	No	27 (52.9%)	120 (94.5%)	
Implant number/FPD	2	42 (82.4%)	58 (45.7%)	0.000 **
	3	9 (17.6%)	69 (54.3%)	
Implant site grafting	Yes	12 (23.5%)	76 (59.8%)	0.000 **
	No	39 (76.5%)	51 (40.2%)	
Follow-up (yrs)	3–5	18 (35.3%)	64 (50.4%)	0.150
	6–10	15 (29.4%)	33 (26.0%)	
	>10	18 (35.3%)	30 (23.6%)	
Edentulism location	MX	18 (35.3%)	73 (57.5%)	0.007 **
	MD	33 (64.7%)	54 (42.5%)	
Implant location	C/IL	3 (5.9%)	12 (9.4%)	0.701
	PM	18 (35.3%)	40 (31.5%)	
	M	30 (58.8%)	75 (59.1%)	
Opposing surfaces	Natural teeth	3 (5.9%)	55 (43.3%)	0.000 **
	IP-FPD (acrylic)	9 (17.6%)	9 (7.1%)	
	Natural teeth-FPD (ceramic)	39 (76.5%)	27 (21.3%)	
	IP-FPD (ceramic)	0 (0.0%)	36 (28.3%)	

** Highly Statistically significant.

**Table 7:** Risk factors for mechanical/technical complications.

	Univariate Analysis				Multivariate Analysis			
Parameters:	Pearson Chi-Squared	*p*	Risk of Technical Complications		Binary Logistic Regression			
			OR/RR	95% CI OR/RR *	B Coef.	OR	95% CI OR	*p*
Age group > 60 yrs	8.903	0.003 **	3.839	1.518 ÷ 9.709	0.172			0.879
History of periodontal disease	58.817	0.000 **	17.569	7.687 ÷ 40.158	3.065	21.429	1.941 ÷ 236.601	0.012 *
Oral hygiene (mPI 2-3)	9.454	0.002 **	4.034	1.581 ÷ 10.297	0.704			0.665
Bruxism	43.671	0.000 **	15.238	5.954 ÷ 38.999	3.231	25.293	2.560 ÷ 249.907	0.006 **
Number of implants (FPD with 2 implants’ support)	19.890	0.000 **	5.552	2.494 ÷ 12.357	2.679	14.567	1.260 ÷ 168.402	0.032 *
Implant site without grafting	19.195	0.000 **	4.843	2.316 ÷ 10.130	1.663			0.090
Mandibular edentulism	7.168	0.007 **	2.478	1.264 ÷ 4.860	1.921	6.831	1.069 ÷ 43.640	0.042 *
Opposing arch	64.025	0.000 **	-	-	1.047	2.850	1.103 ÷ 7.363	0.031 *
Constant					−8.435	0.000		0.000

* Statistically significant, ** Highly Statistically significant.

**Table 8:** Kaplan–Meyer survival analysis results.

	Estimated Average	SEM	95% CI (Average)	
			Lower Limit	Upper Limit
Implant survival/success	16.528	0.189	16.157	16.899
Implants without complications	12.471	0.429	11.631	13.312
Implants without biological complications	15.420	0.387	14.662	16.178
Absence of mechanical/technical complications	13.159	0.439	12.298	14.019

## Data Availability

The data presented in this study are available on request from the corresponding author.
